# Person‐centred care: shifting the power dynamic in the delivery of adolescent and youth‐friendly health services

**DOI:** 10.1002/jia2.26116

**Published:** 2023-07-06

**Authors:** Lynn Phillips, Heleen Soeters, Maximina Jokonya, Allen Kyendikuwa, Luann Hatane

**Affiliations:** ^1^ Paediatric‐Adolescent Treatment Africa (PATA) Cape Town South Africa; ^2^ Global Network of Young People Living with HIV (Y+ Global) Kampala Uganda

1

The provision of healthcare has evolved from applying a traditional, paternalistic, provider‐driven and disease‐focused approach towards one of person‐centred care (PCC), that engages the client in decision‐making, develops client knowledge and fosters self‐care behaviour. Supporting participation along with greater emphasis on a more engaged, equal and beneficial health partnership between healthcare providers and service users is now recognized as one of the pillars of quality healthcare, and a growing body of evidence underpins the beneficial effects of PCC in provider, caregiver and client satisfaction, client health behaviour, quality of life and better healthcare outcomes [1, 2, 3].

The importance of shifting power to the client is only heightened in the long‐term management of chronic diseases [4], prevention and care services of sexually transmitted infections and when delivering services to adolescents and young people living with HIV (AYPLHIV) in their diversity, and other stigmatized, vulnerable and excluded key populations. The principles and strategies of PCC—building trust, being compassionate and respectful of client preferences, needs, values, environment and cultural background—help to address the inequality of AYPLHIV health service experience, support their active involvement, foster autonomy and trust in their healthcare journey.

PCC is not a new concept, yet there is considerable variability in the definition of PCC, with different frameworks and measurements described in the literature [5]. Although there is some consensus on the most important elements (i.e. client as a unique person, client involvement in care, client information, client–clinician communication and client empowerment), PCC conceptualization is blurry, with few practical strategies, hampering its successful implementation, particularly in low‐income African countries. We still meet healthcare providers who think they are already doing PCC when this may not be the case. Stigma, unfriendliness and judgemental provider attitudes remain a reality on the ground, resulting in a reluctance to access sexual reproductive health rights and HIV services, stay in care or adhere to treatment [6].

The long‐standing partnership of Paediatric‐Adolescent Treatment Africa (PATA), an action network of frontline healthcare providers, together with the Global Network of Young People Living with HIV (Y+ Global), provides an opportunity to harness mutual understanding, and build on the combined expertise and insight on successful and practical strategies on how to positively influence the power imbalance inherent in the health provider−AYPLHIV relationship. Our practical recommendations are informed by the regional joint programme implementation of PATA/Y+ Global, and align with the three key domains identified in a recent systematic review of PCC within the context of HIV treatment settings in southern Africa: staffing, service delivery standards and direct client support services [7].

More investment will be needed both for staff capacity‐building and staff composition. In most African countries, healthcare provider educational systems still favour a paternalistic provider patient relationship, shaping the future attitude of caregivers to prioritize biomedical aspects over client preferences and needs. Multiple, ongoing and layered sensitization strategies are required to equip health providers to deliver adolescent‐friendly health services that are person‐centred. Tools that prove to be successful include (1) values clarification training supporting health providers to reflect upon and re‐define their own values; (2) relationship‐building practice sessions for health providers on how to engage more meaningfully; and (3) the identification of PCC champions who lead the process of shifting power and changing entrenched mindsets.

AYPLHIV have first‐hand experience of service gaps and age‐related challenges, and are ideally placed to understand and respond to the needs of their peers and communities. Engaging AYPLHIV as peer supporters working in partnership with the healthcare team has the potential to facilitate a fundamental shift in power structures. Enlisting AYPLHIV whom the system is supposed to serve in the delivery of those services breaks down inequalities, with the potential to challenge often ingrained practices and beliefs. Through the provision of individual or group psychosocial support, counselling and health promotion in the form of teen clubs and adolescent‐friendly safe spaces, peer support has shown to improve retention in care and viral suppression [8]. Effective peer support rests on successful integration into the clinic team, mutually agreed upon divisions of roles and responsibilities that may vary according to local context and healthcare provider appreciation of the value of peer support.

The quality of service delivery can be positively affected through community‐led monitoring assisting health facilities, providers and users in identifying service delivery challenges and developing quality improvement plans that promote a person‐centred standard of care. Again, AYPLHIV have a vital role to play as change agents, and must be included in the design of tools and their application. The PATA/Y+ quality improvement plan process starts with a gap analysis based on PATA's client satisfaction scorecard [9], which provides AYPLHIV and other key populations with the opportunity to assess the perceived quality and accessibility of a range of services, in a self‐reported, anonymous and voluntary manner, and identify potential areas for improvement. Both health facilities and clients are then engaged to jointly review feedback against a matrix of acceptable and comprehensive standards of care, in a productive, collaborative and respectful manner and decide on a matrix of interventions for action.

Client‐centred health system strengthening is possible, but will require institutional commitment and careful implementation across designated roles. An enabling work environment with available resources, including equipment, medication and infrastructure to respond to client needs, will go a long way to increase caregivers’ intrinsic motivation and attitude towards providing nurturing PCC.

## COMPETING INTERESTS

The authors declare that they have no competing interests.

## AUTHORS’ CONTRIBUTIONS

Wrote the paper: LP and LH. Contributed to the writing of the manuscript: HS and MJ. Reviewed the paper: AK. All authors have read and approved the final manuscript.
